# A Nationwide Epidemiological Study of Chronic Kidney Disease Prevalence in a High-Risk Patient Population Without Prior Diagnosis in Primary Health Care in Poland

**DOI:** 10.3390/jcm14103600

**Published:** 2025-05-21

**Authors:** Alicja Jazienicka, Mateusz Babicki, Magdalena Krajewska, Andrzej Oko, Karolina Kłoda, Aleksander Biesiada, Agnieszka Mastalerz-Migas

**Affiliations:** 1 Department of Family Medicine, Wroclaw Medical University, 50-367 Wroclaw, Poland; ma.babicki@gmail.com (M.B.); agnieszka.migas@gmail.com (A.M.-M.); 2 Faculty of Medicine, Wroclaw University of Science and Technology, 50-370 Wroclaw, Poland; magda@softstar.pl; 3 4th Military Teaching Hospital, 53-114 Wroclaw, Poland; 4 Department of Nephrology, Transplantology and Internal Diseases, Poznan University of Medical Sciences, 61-701 Poznan, Poland; aoko@ump.edu.pl; 5 MEDFIT Karolina Kloda, 70-240 Szczecin, Poland; wikarla@gazeta.pl; 6 Polish Society of Family Medicine, 51-141 Wroclaw, Poland; alek.biesiada@gmail.com; 7 Ad Vitam Przychodnia Zdrowia Sp. z o. o. Sp, 32-052 Radziszów, Poland

**Keywords:** chronic kidney disease, epidemiology, Poland, primary care, risk factors

## Abstract

**Background/Objectives:** Chronic kidney disease (CKD) affects 10 to 15% of the world population. Currently, there are no reliable epidemiological data on the prevalence of CKD in Poland. The aim of this study was to determine the prevalence of CKD in the population of high-risk primary health care patients without a prior diagnosis of CKD in the Polish population. **Methods:** This multicenter retrospective study examined 5100 patients from 28 medical institutions. Patients were eligible for the study if they were 60–75 years old or had a history of the risk factors such as hypertension, kidney disease other than CKD, diabetes, cardiovascular disease, or obesity. The study was carried out in two stages, each of which included a determination of serum creatinine levels along with an estimation of eGFR and a determination of albuminuria in a urine sample. **Results:** In total, 5100 patients were included in the final analysis. In the univariate analysis, it was found that as age and body mass index values increased, the risk of developing CKD increased; the same correlation was observed when assessing the presence of hypertension, diabetes, or heart failure. In the multivariate analysis, the negative effects of hypertension, age 60–75 years, diabetes, heart failure, cardiovascular diseases, and kidney disease other than CKD on the development of CKD were confirmed. **Conclusions:** The results indicate that CKD is a significant social problem among the Polish population with risk factors.

## 1. Introduction

Chronic kidney disease (CKD) is considered a significant health problem due to its prevalence. CKD is the result of a decrease in the number of nephrons as a result of a direct destructive process. According to the Kidney Disease Improving Global Outcome (KDIGO) guidelines, CKD is defined as an abnormality in the structure or function of the kidneys that persists for at least three months, leading to health consequences. The disease can be a complication of various nephropathies, hypertension, obesity, and diabetes, and should be listed alongside those as a disease of civilization [1,2,3,4]. Despite the multitude of risk factors, diabetic kidney disease is considered by far the most common cause of CKD [5]. Another crucial risk factor is hypertension, which, due to its convergent pathophysiology, accelerates the onset and progression of CKD [1]. Other known risk factors include older age, obesity, and kidney diseases of other causes, including glomerulonephritis and tubulointerstitial nephritis. The risk of kidney damage is increased by heart failure and other cardiovascular diseases, primarily through pre-renal mechanisms and reduced perfusion [1,3,5].

There are no current data on the prevalence of CKD in Poland. It is estimated that the disease remains undetected in the majority of patients and may affect up to 4 million Polish citizens [1]. In a regional pilot study, PolNef, conducted in 2004 on a population with risk factors, CKD was diagnosed in 11.9% of participants after accounting for albuminuria. Considering other symptoms, as well as pathological urine sediment and changes on ultrasound imaging of the kidneys, the percentage of people with this condition rose to 18% [6]. More optimistic data comes from a cross-sectional and observational study by Natpol, which was conducted in 2011 among Polish patients aged 18–79. According to the analysis, CKD was present in 5.8% of the study population [7]. In 2022 data presented by the Ministry of Health, the prevalence per 100,000 population after standardization was estimated to be 1927, with a death ratio equal to 232 [8]. The prevalence of CKD worldwide varies according to different diagnostic criteria, the capacity of the health system, the age distribution of the population, and risk factors. Currently, considering an estimated glomerular filtration rate (eGFR) of <60 mL/min/1.73 m^2^ as a diagnostic criterion for CKD, the prevalence is estimated at 10–15% worldwide [9,10].

Due to the long course with minimal symptoms, many patients do not see a doctor or undergo tests for the diagnosis of CKD. As a result, the diagnosis is usually made at an advanced stage of the disease, when therapy has limited effectiveness. The early initiation of non-pharmacological and pharmacological treatments improves the prognosis of patients, which is why the vigilance of physicians and an increased detection of the disease in its early stages is so important for the diagnostic and therapeutic process [4,11,12]. The medical specialties that are often in contact with patients in the early stages of the disease and whose diagnostic vigilance seems particularly important are primary care physicians and physicians from other specialties working in primary health care. To raise awareness among primary care physicians, the Polish Society of Family Medicine, together with the Polish Society of Nephrology, designed an epidemiological study. The project additionally involved an educational component aimed at primary care physicians, which preceded participation in the epidemiological study [13].

Due to the lack of recent epidemiological data and a concurrent increasing incidence, the main objective of the project, the results of which are presented below, was to assess the prevalence of CKD in the Polish population among primary healthcare patients at high risk of developing the disease.

## 2. Materials and Methods

A nationwide, multicenter, retrospective epidemiological study of the prevalence of CKD in a high-risk patient population in Poland was conducted. Thirty-seven physicians working in 28 primary care centers participated in the data collection. The study was part of the Chronic Kidney Disease Project.

This epidemiological study was conducted and based on the analysis of medical records. The data was entered into the database by individual family doctors caring for specific patients.

### 2.1. Inclusion Criteria

Inclusion criteria for the study included the following:

- Age 60–75 years (individual criterion even without comorbidities);
- Younger patients meeting at least one of the following conditions:
  ○ A history of kidney disease (other than CKD—structural urinary tract disease, recurrent kidney calculi, drug-induced nephrotoxicity, radiation nephritis, polycystic kidney disease, APOL1-mediated kidney disease, prior acute kidney injury and Alport syndrome);
  ○ A history of diabetes;
  ○ A history of hypertension;
  ○ Cardiovascular disease other than a history of hypertension;
  ○ Obesity.

### 2.2. Exclusion Criteria

Exclusion criteria from participation in the study included the following:

- Age < 18 and >75 years;
- A diagnosis of CKD.

### 2.3. Epidemiological Study Design

In our study, information on age, gender, place of residence, height, weight, and past medical history, including chronic conditions and current medications, was collected.

The study included the patients’ documentation if results were collected in two stages as described below.

The first stage involved performing the following laboratory tests for each patient meeting the inclusion criteria:

- the measurement of serum creatinine levels with an estimation of eGFR and
- the determination of albuminuria in a urine sample.

eGFR was estimated based on the CKD-EPI formula. Albuminuria was tested in a single urine sample.

**Figure 1:**
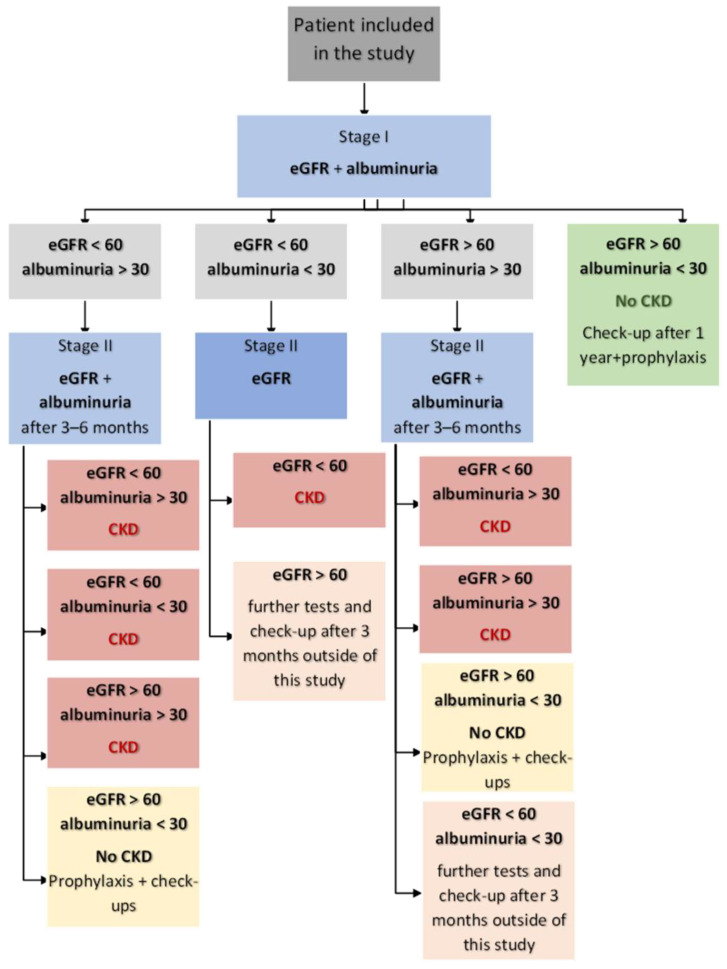
Management algorithm for the study. Albuminuria [mg/g]; eGFR—estimated glomerular filtration rate [mL/min].

Further management depended on the results of the above laboratory tests. Documentation for patients with an eGFR > 60 mL/min/1.73 m^2^ and an albuminuria < 30 mg/g was excluded from the study in the second stage, after the first test showed normal values. Patients with an eGFR < 60 mL/min/1.73 m^2^ and/or an albuminuria > 30 mg/g were required to repeat the tests in 3–6 months. Two results of an eGFR < 60 mL/min/1.73 m^2^ within 3–6 months and/or two findings of albuminuria in a urine sample of >30 mg/g within the same period were considered as the criteria for a diagnosis of CKD. A detailed management algorithm for the documentation used in the epidemiological study is presented in Figure 1.

The study was conducted following the Declaration of Helsinki, and approval for the study was obtained from the Bioethics Committee of the Lower Silesian Medical Board. (Number of approvement: 1/BNR/2022 8 June 2022). Informed consent was not collected due to the retrospective nature of the study and the full anonymization of the patients.

### 2.4. Statistical Analysis

The analyzed variables were qualitative and quantitative. To assess the normality of the distribution, the Shapiro–Wilk Test was performed. To compare the quantitative variables between the two groups, a non-parametric Mann–Whitney test was used. A comparison of the qualitative variables was performed using the chi-square test. To evaluate the correlation between the quantitative variables, the Spearman correlation test was applied. Logistic regression analysis was used to assess the risk factors for the development of CKD. Initially, a univariate analysis was conducted, where the dependent variable was the diagnosis of CKD and the independent variables included sociodemographic data, body mass index (BMI), and specific chronic conditions. In the next step, a multivariate logistic regression model was built, where the dependent variable was the diagnosis of CKD and the independent variables included the same variables as in the previous model.

The level of significance was established at a *p*-value of 0.05 and the analyses were performed using Statistica 13.0 by StatSoft (TIBCO Software, Palo Alto, CA, USA).

## 3. Results

### 3.1. General Characteristics of the Study Group

The final analysis included 5100 patients with a mean age of 60.7 ± 10.6 years. The analyzed group was predominantly female (54.1%) and rural residents (77.2%). Each of the studied patients had at least one risk factor for the development of CKD, the most common of which were hypertension (66.2%) and an age between 60 and 75 years (59.7%). The characteristics of the study group are shown in Table 1.

**Figure 2:**
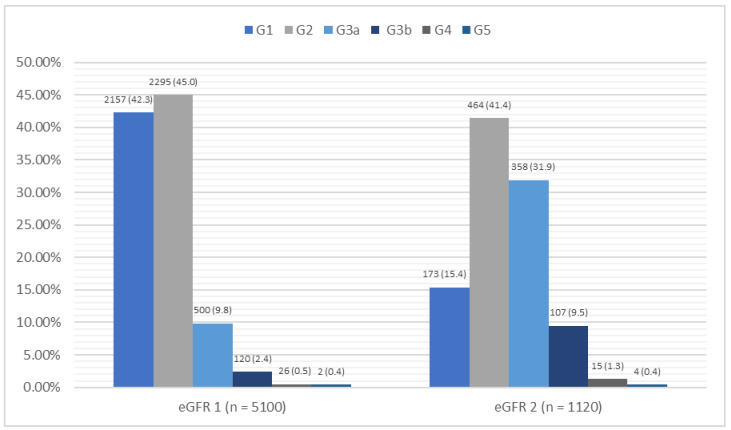
Summary of the eGFR [mL/min/1.73 m^2^] results and diagnostic criteria for CKD in the study group. CKD—chronic kidney disease; eGFR—estimated glomerular filtration rate; m—mean; n—number; SD—standard deviation.

**Figure 3:**
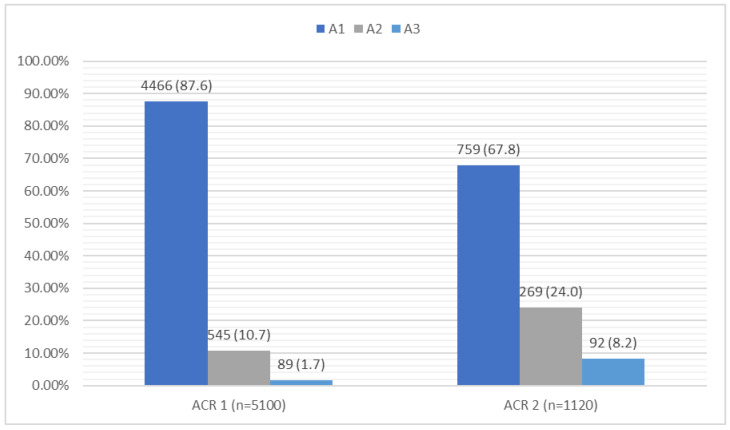
Summary of the ACR [mg/g] results and diagnostic criteria for CKD in the study group. ACR—albumin-to-creatinine ratio; CKD—chronic kidney disease; m—mean; n—number; SD—standard deviation.

In the group of 5100 patients, an abnormal eGFR value (<60 mL/min/1.73 m^2^) was seen in 648 patients representing 13.1% of the study group; two patients had an eGFR < 15 mL/min/1.73 m^2^, indicating renal failure. In the evaluation of the albumin-to-creatinine ratio (ACR), 634 patients (12.4%) had an ACR > 30 mg/g. In the study group, 1120 patients met the inclusion criteria for stage II, i.e., they had an eGFR < 60 mL/min/1.73 m^2^ and/or an ACR > 30 mg/g. Based on the control after 3 months, the criteria for the diagnosis of CKD was met by 724 patients (14.2%), with 448 (8.5%) meeting the criteria based on their eGFR and 347 (6.8%) based on the ACR. A detailed summary of the laboratory results is shown in Figure 2 and Figure 3.

### 3.2. Risk Factors for Developing CKD

#### 3.2.1. Results of Univariate Logistic Regression Analysis

Univariate analysis showed that the risk of developing CKD increased with increasing age and BMI values. Similar observations were made when evaluating patients with hypertension, diabetes, or heart failure. The data also indicated that patients aged 60 to 75 years have a significantly higher risk of developing CKD than those aged <60 years. As the number of analyzed risk factors increases, the risk of developing CKD increases. A detailed summary of the univariate analysis is shown in Table 2.

#### 3.2.2. Results of Multivariate Logistic Regression Analysis

**Figure 4:**
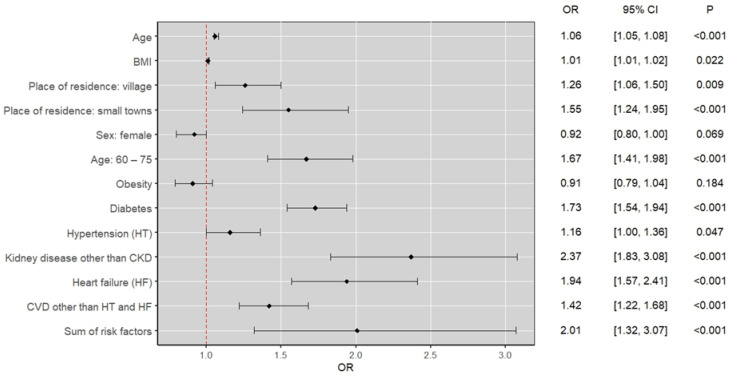
Summary of the results of the multivariate logistic regression analysis.

In the multivariate analysis, the negative impact of hypertension, age 60–75 years, diabetes mellitus, heart failure, cardiovascular conditions, and non-CKD kidney diseases on the development of CKD was confirmed. In addition, it was shown that as the number of specific risk factors increases, the risk of developing CKD increases. A detailed summary is shown in Table 3. A summary of the results of the multivariate logistic regression analysis is presented in Figure 4.

## 4. Discussion

To the best of the authors’ knowledge, this is the first study in the Polish population with risk factors for CKD conducted in recent years to investigate the prevalence of this disease. In recent months Masajtis-Zagajewska et al. published a study showing that the clinical and economic burden of patients with CKD will worsen in the upcoming years, and that policies to enhance the early detection of CKD and the initiation of treatment to slow the disease progression should be implemented [14].

In our epidemiological study, the prevalence of CKD in the Polish population with risk factors was 14.2%. Only patients with the risk factors described in the methodology section were included in the study. In 8.5% of patients who qualified for the study, a diagnosis of CKD was made based on an abnormal eGFR value determined twice at an interval of at least 3 months, and in 6.8% based on the ACR determined over the same period. Our results on the prevalence of CKD corresponded with the data obtained in other global and European studies. In a large meta-analysis including a review of the literature and databases from almost 200 countries around the world, the global prevalence of CKD in the general population was estimated to be 9.5% (interquartile range IQR 5.9–11.7). The highest prevalence of CKD was observed in eastern and central Europe at 12.8%. The overall prevalence appears to be underestimated in the African continent, where CKD was diagnosed in only 4.2% of patients. This result, which is probably incomplete, differs significantly from data obtained from the rest of the world, where the disease has been diagnosed in 12.2–15.8% of study participants [15,16]. Other meta-analyses have estimated the prevalence of CKD among the African population as being at a similar level to data obtained from other continents [17]. According to statistical reviews (2008, 2013, 2022), the prevalence of CKD was estimated at 7.2–16% of the general population worldwide, depending on age group and place of residence [18,19,20].

The differences in the prevalence of CKD in different geographic areas are partly due to the diagnostic methods used, but also largely depend on the racial and ethnic influences and the prevalence of CKD risk factors in the population [21]. The results of an analysis by Brück et al., including data from 19 general population studies from 13 European countries, showed that among the European population aged 20–74 years, the CKD prevalence ranged from 3.31% in patients from Norway to 17.3% in patients from northern Germany [10]. However, the cited studies determined the prevalence of CKD in the general population, not in populations with specific risk factors, so they cannot be compared directly to our results.

Andong Ji et al. conducted a large epidemiological study on the prevalence of CKD among an elderly patient population living in China. In the study, CKD was detected in 11.4% of patients. It was shown that the risk of developing CKD increased with age and the presence of hypertension, diabetes, and obesity. These findings are consistent with our results. However, the mean age in the Chinese population studied was 71 years, while in our study group it was less than 61 years. This is the main feature that differentiates the two analyzed populations [22].

The cross-sectional observational NATPOL study conducted in 2011, designed to assess the prevalence of cardiovascular risk factors in Poland, evaluated the prevalence of CKD among adult Polish citizens. The prevalence of CKD (based on the Chronic Kidney Disease Epidemiology Collaboration formula) was estimated at 5.8%, and the prevalence of albuminuria was estimated at 4.5%. The study included a group of patients aged 18–79 years, not just those with a history of risk factors, which may explain the lower prevalence of the disease found in the data obtained [7,23]. A much higher prevalence of CKD was seen in the PolNef study conducted in the Pomeranian province. In that study, the presence of CKD at various stages was found among 18.4% of adult patients. It is noteworthy, however, that elderly patients and those with multimorbidities were enrolled in the PolNef program, as evidenced by the fact that 68% of the subjects declared that they were constantly under the care of physicians from various specialties [6].

Although the examination of microalbuminuria in a urine sample is non-invasive and valuable for both predicting the risk of renal dysfunction and monitoring the effect of treatment, it is still used sporadically in daily medical practice. Several global reports indicate a low rate of albuminuria measurement in patients with type 2 diabetes, an indicator that is underutilized in the monitoring of CKD by primary care practitioners in the United States and Denmark, according to the available data [9,24,25].

In the present study, an abnormal ACR measurement recorded in two samples at least 3 months apart mandated a diagnosis of CKD despite a normal eGFR in 279 patients. This demonstrates how important microalbuminuria is as a parameter in the diagnosis of renal dysfunction. The ability of primary care physicians to order the ACR test during follow-up blood tests has the potential to improve the detection of CKD in a population with risk factors. The test can be performed as part of the recently introduced Coordinated Care Program in Poland’s healthcare system. It aims to provide comprehensive care by primary care physicians to patients burdened with the most common chronic disease entities, including CKD. Primary care physicians working in facilities implementing the cardiology, diabetes, and nephrology pathways under the Coordinated Care Program can order ACR tests free-of-charge for their patients [26].

The data analyzed show that as age and BMI increases, the risk of developing CKD increases. CKD is more common in patients with hypertension, diabetes, and heart failure. It has also been shown that patients aged between 60 and 75 years have a significantly higher risk of developing CKD than those aged <60 years. As the number of risk factors present in a patient increases, the risk of developing CKD increases. Glomerulonephritis, which has historically been one of the most common causes of CKD, has receded due to an increase in the prevalence of diseases of civilization [4].

Similar results were obtained in the NatPol study. Insulin-dependent diabetes and hypertension were the two most important risk factors for developing CKD, in addition to older age [7]. A 2016 meta-analysis including 100 studies of varying quality involving 6,908,440 patients shows that the incidence of CKD increases with age [27].

Numerous studies confirm the link between an increased BMI and the deterioration of renal function. Obesity leads to CKD directly as an independent risk factor, and indirectly by increasing the chance of other risk factors such as diabetes, hypertension, and atherosclerosis. An increased BMI leads indirectly to changes in renal hemodynamics, hormones, and local growth factors, causing hyperfiltration damage and focal segmental glomerular sclerosis [28]. A 2022 international study by Zheng J et al. [29] considers an increase in the BMI above 25 kg/m^2^ to be among eight reliable risk factors for CKD in the European population and four reliable risk factors for CKD in the Asian population. The study’s authors also showed an increased risk of developing CKD in patients with hypertension and type 2 diabetes, which corresponds with the data obtained in our study. Other risk factors reported for the European population include high systolic blood pressure, abnormal high density lipoprotein (HDL) cholesterol, apolipoprotein A-I, and lipoprotein levels, and urolithiasis [29].

The pathophysiology of hypertension is closely linked to disorders that lead to the development of CKD such as endothelial dysfunction, deregulation of sodium metabolism, activation of the sympathetic nervous system, and increased activity in the renin–angiotensin–aldosterone system [30,31]. Our study showed that hypertension is a risk factor predisposing to the development of CKD, similar to data available from other studies [32].

According to the available literature, CKD associated with diabetes is the most common cause of end-stage renal disease [33,34]. Both type 1 and type 2 diabetes can cause progressively increasing microvascular and macrovascular complications that contribute to the development of CKD. In addition, the long asymptomatic course of type 2 diabetes affects the diagnosis and implementation of appropriate management, often at a later stage of the disease when multi-organ complications including renal dysfunction develop [35]. In a study performed in Nepal among patients with type 2 diabetes, CKD was detected in more than 85% of the study participants [36].

According to a large observational study, the Third National Health and Nutrition Examination Survey (NHANES III), there was a strong correlation between the presence of CKD and type 2 diabetes and an increased risk of mortality. In that study, the authors observed a mortality rate of more than 31% in patients with CKD and diabetes, compared with 11.5% in those with diabetes alone. An observational study using linked databases from the United States and the United Kingdom found that the presence of both CKD and type 2 diabetes correlated with an increased risk of major adverse cardiac events [34].

CKD is one of the most serious sequelae of heart failure due to several common pathomechanisms arising from inflammatory and immunological responses. Decreased perfusion caused by a low cardiac output and venous stasis is primarily responsible for the deterioration of renal function. In addition, renal insufficiency and impaired salt and water excretion result in increased cardiac preload and afterload, which leads to the exacerbation of heart failure in a vicious cycle [37,38]. A large meta-analysis by Kevin Damman et al. in 2013 reported that the above diseases are closely related [39]. Our results also indicate that heart failure is a risk factor for developing CKD.

The authors are aware of the limitations of the conducted study. First, it is worth noting that the study group consisting of 5100 patients is quite limited for an epidemiological study. We realize that the studied group of patients does not accurately reflect the gender and residence distribution of the Polish population, but it is similar. In the study population, the percentages of women and men were approximately 54% and 46%, respectively, while in the Polish population it is 52% and 48%, respectively [40]. A greater discrepancy is observed in the residence distribution of patients included in this study. The 2021 census included data indicating that urban residents account for almost 60% of the Polish population, and rural residents account for 40%, which deviates quite significantly from the population of patients included in this epidemiological study [41]. This study analyzed the prevalence of CKD among patients with risk factors, so the results cannot be directly related to the general Polish population. The study did not include a control group consisting of patients without chronic diseases, with a normal BMI, and younger than 60 years of age.

### Clinical Implications

Taking into consideration that chronic kidney disease is a common condition in the

population, especially in patients with risk factors, every physician, especially family medicine physicians, should be vigilant in detecting it. ACR testing is very important in diagnosing CKD and should be performed in addition to eGFR testing. In Poland this has been made easier by the introduction of a Coordinated Care Model that does not require the patient to contribute any costs towards financing this test. Patients aged 60–75 years should be particularly carefully screened for chronic kidney disease even in the absence of risk factors. Due to the long asymptomatic course, a proactive physicians’ attitude and the monitoring of patients even in the absence of symptoms seems to be extremely important. Owing to common pathomechanisms, CKD coexists with other civilization diseases that should be taken into account in control tests.

## 5. Conclusions

This epidemiological study presents the current prevalence of CKD in a population with risk factors in Poland. The criteria for the diagnosis of CKD were met by 14.2% of patients, of whom 8.5% met the criteria based on the eGFR, and 6.8% met the criteria based on the ACR. Considering the obtained data, CKD is a significant health problem in Poland. As in other European countries, Poland’s aging population is burdened with numerous risk factors, such as obesity, hypertension, diabetes, and heart failure, leading to the development of CKD. The screening and early diagnosis of CKD are possible in Polish primary care, especially with the introduction of the new Coordinated Care Model. The treatment and monitoring of patients in the early stages of CKD can be carried out initially by the primary care physician and in later stages in cooperation with a nephrologist. In the future, we plan to examine the relationship between the use of individual medications taken by patients in our study group and the development of CKD.

## Figures and Tables

**Table 1:** Characteristics of the study group.

Variables		The Whole Group N (%) M ± SD	CKD N (%) M ± SD	Non-CKD N (%) M ± SD	*p*
Age		60.7 ± 10.6	64.5 ± 9.1	60.1 ± 10.7	<0.001
BMI		29.9 ± 5.6	30.5 ± 5.6	29.9 ± 5.6	0.004
Sex	Female	2759 (54.1)	384 (13.9)	2375 (86.1)	0.537
	Male	2341 (45.9)	340 (14.5)	2001 (85.5)	
Place of residence	Village	3937 (77.2)	557 (14.2)	3380 (85.8)	0.068
	Up to 500 thousand	511 (10.0)	87 (17.0)	424 (83.0)	
	>500 thousand	652 (12.8)	80 (12.3)	572 (87.7)	
Risk factors for CKD	Age 60–75	3046 (59.7)	508 (16.6)	2558 (83.4)	<0.001
	Obesity	1894 (37.1)	277 (14.6)	1617 (85.4)	0.499
	Diabetes	1041 (20.4)	232 (22.3)	809 (77.7)	<0.001
	Hypertension	3377 (66.2)	526 (15.6)	2851 (84.4)	<0.001
	Kidney disease other than CKD	271 (5.3)	36 (13.3)	235 (86.7)	0.656
	Heart failure	129 (2.5)	48 (37.2)	81 (62.8)	<0.001
	Cardiovascular diseases other than hypertension and heart failure	350 (6.9)	96 (27.4)	254 (72.6)	<0.001
	The sum of risk factors	2.0 ± 1.1	2.4 ± 1.2	1.9 ± 1.0	<0.001

BMI—Body Mass Index; CKD—Chronic Kidney Disease; M—mean; N—number; *p*—statistical significance; SD—standard deviation.

**Table 2:** Univariate logistic regression analysis of the effect of specific risk factors on the development of CKD in the study group.

Variable	OR [95Cl]	*p*
Age, years	1.04 [1.03, 1.06]	<0.001
BMI	1.01 [1.01, 1.03]	0.007
Place of residence, village	1.17 [0.92, 1.52]	0.773
Place of residence, <500 thousand residents	1.47 [1.06, 2.04]	0.024
Sex, female	1.05 [0.89, 1.23]	0.536
Age 60–75	1.67 [1.41, 1.98]	<0.001
Obesity	1.05 [0.89, 1.24]	0.499
Diabetes	2.08 [1.75, 2.47]	<0.001
Hypertension	1.42 [1.19, 1.69]	<0.001
Kidney disease other than CKD	0.92 [0.64, 1.32]	0.658
Heart failure	3.76 [2.61, 5.43]	<0.001
Cardiovascular diseases other than hypertension and heart failure	2.47 [1.93, 3.18]	<0.001
The sum of risk factors	1.58 [1.53, 1.63]	<0.001

BMI—body mass index; CKD—chronic kidney disease; OR—odds ratio; *p*—statistical significance.

**Table 3:** Summary of the results of the multivariate logistic regression analysis.

Variable	OR [95Cl]	*p*	R^2^ Nagelkerke
Age, years	1.06 [1.05, 1.08]	<0.001	0.099
BMI	1.01 [1.01, 1.02]	0.022	
Place of residence, village	1.26 [1.06, 1.50]	0.009	
Place of residence, <500 thousand residents	1.55 [1.24, 1.95]	<0.001	
Sex, female	0.92 [0.80, 1.00]	0.069	
Age 60–75	1.67 [1.41, 1.98]	<0.001	
Obesity	0.91 [0.79, 1.04]	0.184	
Diabetes	1.73 [1.54, 1.94]	<0.001	
Hypertension	1.16 [1.00, 1.36]	0.047	
Kidney disease other than CKD	2.37 [1.83, 3.08]	<0.001	
Heart failure	1.94 [1.57, 2.41]	<0.001	
Cardiovascular diseases other than hypertension and heart failure	1.42 [1.22, 1.68]	<0.001	
The sum of risk factors	2.01 [1.32, 3.07]	<0.001	

BMI—body mass index; CKD—chronic kidney disease; OR—odds ratio; *p*—statistical significance.

## Data Availability

The data presented in this study are available on request from the corresponding author.

## Abbreviations

The following abbreviations are used in this manuscript:

CKD	Chronic kidney disease
ACR	Albumin-to-creatinine ratio
eGFR	Estimated glomerular filtration rate
BMI	Body mass index
OR	Odds ratio
P	Statistical significance
SD	Standard deviation
